# The Kidney Failure Risk Equation for prediction of end stage renal disease in UK primary care: An external validation and clinical impact projection cohort study

**DOI:** 10.1371/journal.pmed.1002955

**Published:** 2019-11-06

**Authors:** Rupert W. Major, David Shepherd, James F. Medcalf, Gang Xu, Laura J. Gray, Nigel J. Brunskill

**Affiliations:** 1 Department of Health Sciences, University of Leicester, Leicester, United Kingdom; 2 John Walls Renal Unit, Leicester General Hospital, University Hospitals of Leicester NHS Trust, Leicester, United Kingdom; 3 Kettering General Hospital, Kettering, United Kingdom; 4 Department of Cardiovascular Sciences, University of Leicester, Leicester, United Kingdom; University of Oxford, UNITED KINGDOM

## Abstract

**Background:**

The Kidney Failure Risk Equation (KFRE) uses the 4 variables of age, sex, urine albumin-to-creatinine ratio (ACR), and estimated glomerular filtration rate (eGFR) in individuals with chronic kidney disease (CKD) to predict the risk of end stage renal disease (ESRD), i.e., the need for dialysis or a kidney transplant, within 2 and 5 years. Currently, national guideline writers in the UK and other countries are evaluating the role of the KFRE in renal referrals from primary care to secondary care, but the KFRE has had limited external validation in primary care. The study’s objectives were therefore to externally validate the KFRE’s prediction of ESRD events in primary care, perform model recalibration if necessary, and assess its projected impact on referral rates to secondary care renal services.

**Methods and findings:**

Individuals with 2 or more Chronic Kidney Disease Epidemiology Collaboration (CKD-EPI) eGFR values < 60 ml/min/1.73 m^2^ more than 90 days apart and a urine ACR or protein-to-creatinine ratio measurement between 1 December 2004 and 1 November 2016 were included in the cohort. The cohort included 35,539 (5.6%) individuals (57.5% female, mean age 75.9 years, median CKD-EPI eGFR 51 ml/min/1.73 m^2^, median ACR 3.2 mg/mmol) from a total adult practice population of 630,504. Overall, 176 (0.50%) and 429 (1.21%) ESRD events occurred within 2 and 5 years, respectively. Median length of follow-up was 4.7 years (IQR 2.8 to 6.6). Model discrimination was excellent for both 2-year (C-statistic 0.932, 95% CI 0.909 to 0.954) and 5-year (C-statistic 0.924, 95% 0.909 to 0.938) ESRD prediction. The KFRE overpredicted risk in lower (<20%) risk groups. Reducing the model’s baseline risk improved calibration for both 2- and 5-year risk for lower risk groups, but led to some underprediction of risk in higher risk groups. Compared to current criteria, using referral criteria based on a KFRE-calculated 5-year ESRD risk of ≥5% and/or an ACR of ≥70 mg/mmol reduced the number of individuals eligible for referral who did not develop ESRD, increased the likelihood of referral eligibility in those who did develop ESRD, and referred the latter at a younger age and with a higher eGFR. The main limitation of the current study is that the cohort is from one region of the UK and therefore may not be representative of primary care CKD care in other countries.

**Conclusions:**

In this cohort, the recalibrated KFRE accurately predicted the risk of ESRD at 2 and 5 years in primary care. Its introduction into primary care for referrals to secondary care renal services may lead to a reduction in unnecessary referrals, and earlier referrals in those who go on to develop ESRD. However, further validation studies in more diverse cohorts of the clinical impact projections and suggested referral criteria are required before the latter can be clinically implemented.

## Abstract 2

In this cohort study, Rupert Major and colleagues evaluate the performance of the Kidney Failure Risk Equation in chronic kidney disease patients in primary care settings and project impact of a recalibrated equation on referral practices and patient outcomes.

## Author summary

**Why was this study done?:**

- The Kidney Failure Risk Equation (KFRE) has been developed in multinational chronic kidney disease (CKD) cohorts to predict end stage renal disease (ESRD) events (the need for dialysis or a kidney transplant) over 2- and 5-year time periods.
- The performance of the KFRE has not been extensively tested in UK and European primary care CKD populations.
- The projected impact of the KFRE on referrals from primary to secondary care has not been studied, and it is unclear what criteria for referrals should be used.

**What did the researchers do and find?:**

- Our study suggests that the KFRE has largely accurate performance (discrimination) for predicting ESRD, but requires adjustment (recalibration) for a primary care setting.
- Compared to current referral criteria, using hybrid criteria of a ≥5% risk of ESRD over 5 years from the recalibrated KFRE and/or a urine albumin-to-creatinine ratio (ACR) of ≥70 mg/mmol would reduce the number of individuals eligible for referral without increasing the number who later develop ESRD and are not initially eligible for referral.

**What do these findings mean?:**

- The recalibrated KFRE can be used to predict the risk of ESRD over 2 and 5 years in primary care CKD.
- The KFRE, in conjunction with the ACR criterion, can be used to triage CKD referrals from primary to secondary care.

## Introduction

Chronic kidney disease (CKD) is a worldwide public health issue and is associated with increased risk of all-cause mortality, cardiovascular disease, and end stage renal disease (ESRD) (the need for dialysis or renal transplantation) [1–4]. CKD care is estimated to cost the National Health Service (NHS) in England around £1.3 billion per annum, of which £780 million relates to ESRD [5]. However, CKD may be the most common non-cancer condition subject to ‘overdiagnosis’, with its associated psychological and financial costs of disease labelling, repetition of investigations, and overtreatment [6,7]. Many individuals with CKD will be at low risk for progression to ESRD, but will have raised risk of cardiovascular disease events. The introduction of routine reporting of estimated glomerular filtration rate (eGFR) and the development of primary care CKD registers approximately 15 years ago may have increased secondary care renal referrals in the UK without a clear benefit [8].

Clinical risk prediction models aim to estimate the risk of an event for an individual using their related information. The 3 main purposes of risk prediction models are to aid treatment decisions and prognostication in clinical practice; to assist research planning, particularly in relation to clinical trials; and to assess healthcare systems and resource management [9]. Risk models appear regularly, but few are externally validated in other populations or have their potential impact studied [10]. Updating a risk prediction model through processes such as recalibration is common and is likely to improve a model’s performance in different geographical and temporal settings [11]. There have been more than 350 models developed to predict cardiovascular disease risk, but few have had independent assessment of their performance or projected impact [12]. A 2011 systematic review identified 11 ESRD prediction models, each of which had significant limitations, including the lack of external validation and limited clinical utility [13].

Subsequently, prediction tools for ESRD have been developed by meta-analysis of individual participant data from 31 cohorts of predominately North American CKD populations [14]. Three ESRD prediction equations, based on 4, 6, or 8 variables, were derived and assessed. All included the variables of age, sex, eGFR, and urine albumin-to-creatinine ratio (ACR) (4-variable), with the addition of diabetes mellitus and hypertension (6-variable) or serum albumin, bicarbonate, calcium, and phosphate (8-variable). The performance of the 4-variable Kidney Failure Risk Equation (KFRE) was similar to that of the other 2 equations and on the basis of parsimony was recommended as the model for implementation into clinical practice [14]. However, overall (‘baseline’) risk was found to be lower in non–North American cohorts, and therefore adjustment of risk for this population using a calibration factor was proposed [14]. Evaluation of the role of the KFRE in patient referral from primary to secondary care has been highlighted as an aim for updating the CKD National Institute for Health and Clinical Excellence (NICE) guidelines [15]. However, of the 31 cohorts studied only 2, GLOMMS-1 and CRIB, were UK-based, both comprising secondary care patients [14]. Both UK cohorts had lower average eGFR, higher prevalence of proteinuria, and higher prevalence of primary renal disease, such as glomerulonephritis, than is typically seen in primary care CKD. In addition, their baseline data were collected in the mid-1990s and early 2000s.

The KFRE has been suggested as a triage tool for CKD referrals from primary to secondary care, with a 5-year risk of ESRD of ≥3% or ≥5% suggested as a referral criterion [16]. However, the relevance of the KFRE to a contemporary cohort representative of the UK primary care CKD population is currently unknown, and projections of its implementation have not been studied in this group of patients.

Therefore, the aim of the current study was 3-fold: first, to independently, externally validate the performance of the 4-variable KFRE in an unselected UK primary care CKD population for prediction of ESRD at 2 and 5 years; second, to assess the need for recalibration in the same population; and third, to perform a clinical impact projection study assessing how implementing the KFRE in primary care would affect CKD referrals to secondary care.

## Methods

A primary care CKD study cohort was established from general practices using searches of clinical electronic patient records for CKD-relevant clinical data (extracted between 1 December 2009 and 1 November 2011) for a study time period starting 1 December 2004. Follow-up was continued until 1 November 2016, and the associated data extraction occurred between 7 September 2010 and 13 March 2018. All practices were based in 4 Clinical Commissioning Groups (CCGs), local groupings of primary care practices. The CCGs were East Leicestershire and Rutland CCG, Leicester City CCG, Nene CCG (Northamptonshire), and West Leicestershire CCG. All data were anonymised prior to transfer to the research database. Written informed consent for use of the data was received from the individual general practices. Ethical approval was received from the East Midlands (Leicester Central) Research Ethics Committee (09/H0406/117 and 16/EM/0315). Patients and public were involved during the application process for the grants that funded the cohort. Individual patient consent was waived by the ethics committee as all data were anonymised.

The initial inclusion criteria for the CKD-related data searches were based on Modification of Diet in Renal Disease (MDRD) eGFR, the standard for identifying CKD when the cohort was established [17]. For Nene CCG, eligible individuals had a single eGFR < 60 ml/min/1.73 m^2^, and for the 3 Leicestershire CCGs, a single eGFR < 65 ml/min/1.73 m^2^. As the eGFR variable used in the KFRE is based on the Chronic Kidney Disease Epidemiology Collaboration (CKD-EPI) eGFR formula, all serum creatinine results associated with the MDRD eGFRs were used to calculate CKD-EPI eGFRs [18]. For inclusion in the current analysis, individuals were required to have 2 CKD-EPI eGFR values < 60 ml/min/1.73 m^2^ more than 90 days apart and a recorded quantifiable urine proteinuria (ACR or protein-to-creatinine ratio) measurement [19]. The date of the proteinuria measurement became the date for estimating baseline risk using the KFRE and for beginning the follow-up period. The study’s sample size was based on identifying at least 200 ESRD events, to provide accurate and precise estimates of KFRE performance in UK primary care [20].

The Leicester Renal Network records data for the areas of Leicestershire, Rutland, Northamptonshire, North Cambridgeshire, and Lincolnshire. These data are utilised by the UK Renal Registry for identifying ESRD events. Coded ESRD outcomes, including renal transplantation, were identified from both primary and secondary care data. Secondary care renal services data for demographics, eGFR, and proteinuria were also linked from the Leicester Renal Network electronic patient record to the primary care data.

No specific prospective analysis protocol was made. Analysis was planned when NICE indicated in its CKD surveillance report in 2017 that it was evaluating the role of the KFRE for an update to its guidelines [15]. No specific data-driven changes were made; existing evidence was used for the study, such as the KFREs and the risk thresholds tested for referral criteria, including the pre-existing NICE guideline referral criteria of eGFR < 30 ml/min/1.73 m^2^ or ACR ≥ 70 mg/mmol.

Predicted 2- and 5-year ESRD risk was initially calculated using the published KFRE 4-variable non–North American equation (S1 Text). These predicted risks were then used to assess the discrimination performance of the KFRE. Model discrimination describes a model’s performance in separating individuals who experience an event from those who do not. Discrimination was initially assessed using Kaplan–Meier survival curves for the cohort based on the previously proposed risk groups of <3%, 3% to <5%, 5% to <15%, 15% to <25%, 25% to <50% and ≥50% risk for 5-year prediction of ESRD [14]. Discrimination assessment was also performed using Harrell’s C-statistic and Somers’ D, both of which assess the ranking of individuals based on estimated risk and the event of interest.

Calibration was assessed by calculating the beta coefficient of the linear predictor and plotting predicted risk versus observed risk for ESRD. Calibration assessment of a risk prediction model describes how well predictions match observed outcomes. The beta coefficient of the linear predictor gives a global overview of whether a model to be validated is under- or overfitted in the validation data. Plotting predicted versus observed risk gives a visual depiction of whether predictions match what has occurred in the cohort. It is more sensitive to detecting miscalibration of risk within spectra of risk, such as ‘low’ or ‘high’ risk groups, but can be affected by the selection of risk groups within the plot. Therefore, groups for observed ESRD events were based on deciles for the whole cohort and additional 1%-wide risk groups for risk < 10%. Sensitivity analyses of the risk groups selected were performed by performing calibration assessments also based on groups with equal numbers of ESRD events and previous described risk groups [14,16].

Where calibration of the model for 2- or 5-year predicted risk was suboptimal, recalibration was performed by adjusting the baseline risk in the KFRE model. The baseline risk describes the model’s predicted risk when all variables in the model are set as 0. In the KFRE, these values were centred around the mean values in the development cohort. The adjusted baseline risk for recalibration was calculated through post-estimation prediction of the survival function from the cohort’s data.

These data were then used to assess current NICE guidelines for referral to secondary care renal services (CKD-EPI eGFR < 30 ml/min/1.73 m^2^ and/or ACR ≥ 70 mg/mmol) versus potential criteria for referral based on KFRE predicted risk. We initially performed a comparison of numbers of patients recommended for referral at baseline based on NICE criteria [19] and previously proposed criteria for referral using KFRE-calculated 5-year ESRD risk of ≥3%, ≥5%, and ≥15% [14,16]. This analysis included calculations of sensitivity, specificity, numbers of cases of ESRD not referred at baseline, and mean characteristics of referrals for different referral criteria using the KFRE. In order to assess the impact of new referrals to secondary care renal services, for this part of the analysis we excluded all individuals already referred within 2 years of their inclusion in the cohort. Data describing referrals to secondary care renal services were only available for practices based in the 3 Leicester CCGs, and therefore this analysis was based on these practices only (“eligibility assessment cohort”). All data have been reported in line with the TRIPOD statement (S1 Table) [21]. All statistical analysis was performed using Stata version 15.0. Data to reproduce the analysis are available in S1 Data and on Figshare [22].

## Results

The CKD cohort was identified from an adult population of 630,504 individuals registered at 93 general practices. Of these, 35,539 (5.6%) had ≥2 CKD-EPI eGFR values < 60 ml/min/1.73 m^2^ >90 days apart and a recorded ACR and formed the CKD cohort. Baseline demographics for the current CKD cohort compared to the 2 UK-based CKD cohorts used in the KFRE development (UK-KFRE cohorts) [14] are shown in Table 1. Patients were older (mean 7.7 years older, 95% CI 7.6 to 7.8 years, *p* < 0.001) and mean CKD-EPI eGFR was 20.0 ml/min/1.73 m^2^ higher (95% CI in 19.9 to 20.1, *p* < 0.001 for log transformed values) in the current CKD cohort compared to the UK-KFRE cohorts. Overall, 176 and 429 ESRD events occurred respectively within 2 and 5 years of follow-up.

**Table 1:** Baseline characteristics, follow-up, and outcomes of the cohort compared to 2 previous UK-based cohorts.

Variable	Current CKD cohort	UK-KFRE cohorts*
**Baseline characteristics**		
*n*	35,539	1,315^†^
Dates of baseline data	2004–2016	1996–1998 and 2003^‡^
Female	20,436 (57.5%)	757 (45.5%)
Mean age, years	75.9 (SD 10.6)	68.2
Mean CKD-EPI eGFR, ml/min/1.73 m^2^	48.2 (SD 9.8)	28.2
Median CKD-EPI eGFR, ml/min/1.73 m^2^	51 (IQR 43 to 56)	—
Mean ACR, mg/mmol	11.8 (SD 40.9)	—
Median ACR, mg/mmol	3.2 (IQR 1.2 to 8.0)	—
Participants with albuminuria^§^	17,546 (49.4%)	960 (69.1%)
Cardiovascular disease	11,376 (32.0%)	—
Heart failure	3,191 (9.0%)	—
Hypertension	24,833 (69.9%)	988 (71.1%)
Diabetes	11,193 (31.5%)	670 (48.2%)
**Follow-up and outcomes**		
Mean follow-up, years	4.8 (SD 2.5)	—
Median follow-up, years	4.7 (IQR 2.8 to 6.6)	—
Mean time to ESRD, years	3.5 (SD 2.3)	—
Median time to ESRD, years	3.3 (IQR 1.7 to 5.0)	—
ESRD events within 2 year	176	—
ESRD events within 5 years	429	312
ESRD rate, per 1,000 person-years	3.4 (95% CI 3.1 to 3.6)	55.9
Death rate, per 1,000 person-years	55.9 (95% CI 54.8 to 57.0)	—

Data are *n* (percent) unless otherwise indicated.

*UK-based cohorts CRIB and GLOMMS-1 [14].

^†^Overall, 308 of the 382 individuals in CRIB were used in the development of the 4-variable KFRE.

^‡^Dates refer to CRIB and GLOMMS-1, respectively.

^§^Defined in KFRE development cohort as ACR ≥ 30 mg/g (≥3.39 mg/mmol).

ACR, albumin-to-creatinine ratio; CKD, chronic kidney disease; CKD-EPI, Chronic Kidney Disease Epidemiology Collaboration; eGFR, estimated glomerular filtration rate; ESRD, end stage renal disease; KFRE, Kidney Failure Risk Equation.

### Model discrimination

**Fig 1:**
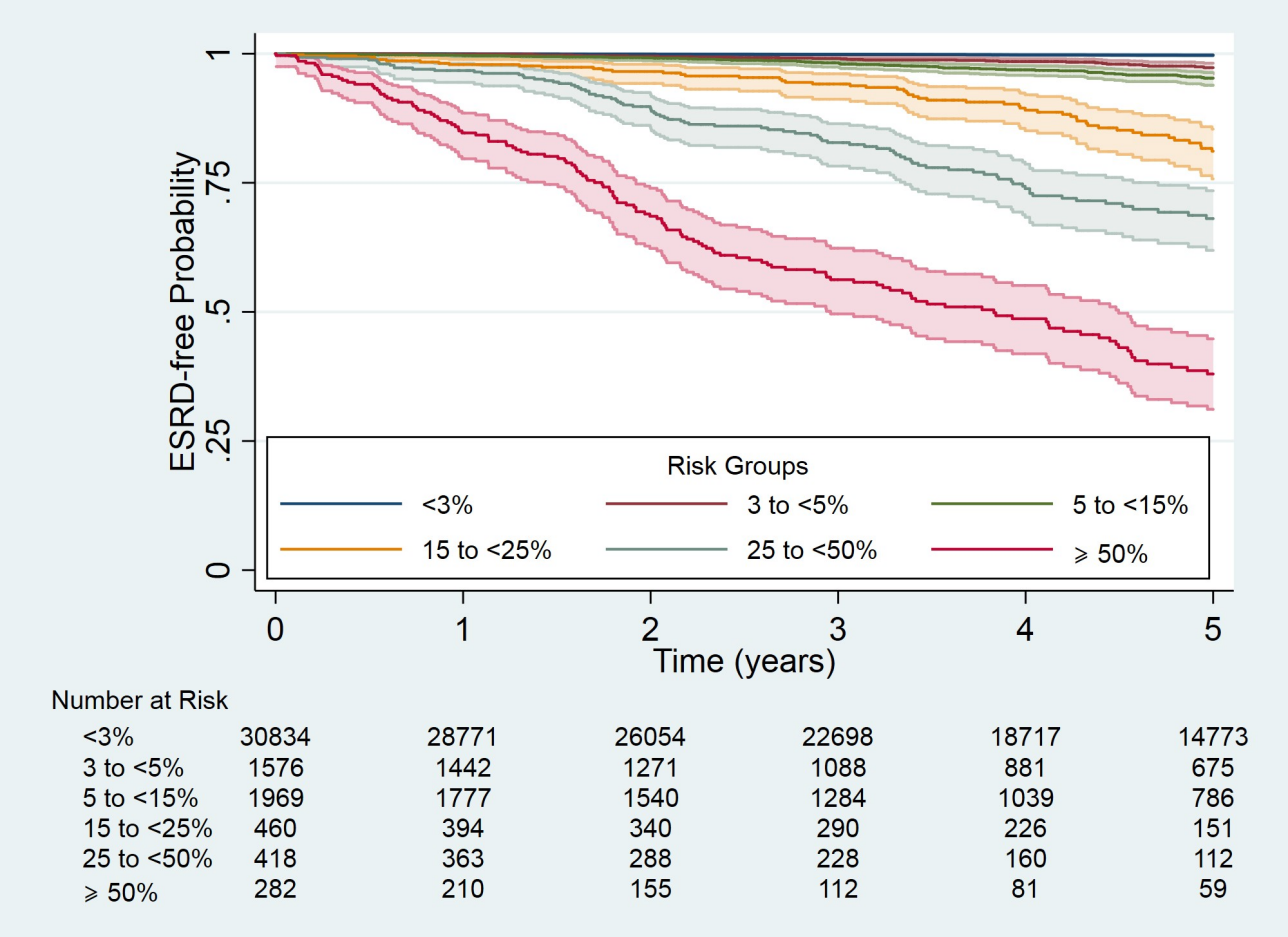
Kaplan–Meier plot of time to ESRD event by risk group. Risk categorisation was based on KFRE-calculated risk at the study’s baseline. Shaded areas represent the 95% confidence intervals for the groups. ESRD, end stage renal disease; KFRE, Kidney Failure Risk Equation.

KFRE discrimination at 5-year follow-up showed good separation of risk for ESRD based on the previously proposed risk categorisations (Fig 1). A summary of KFRE performance in predicting ESRD events at 2 and 5 years is shown in Table 2. Discrimination was excellent for the 4-variable KFRE, with the C-statistic being similar to the development cohort’s pooled C-statistic.

**Table 2:** Summary of KFRE model performance for ESRD events at 2 and 5 years.

Performance measure	2 years	95% CI	5 years	95% CI
C-statistic	0.933	0.910 to 0.956	0.926	0.911 to 0.942
Somers’ D	0.866	0.819 to 0.912	0.853	0.822 to 0.884
Linear predictor β coefficient	1.137	1.057 to 1.216	1.080	1.031 to 1.129
Baseline risk–original model	0.9832		0.9365	
Baseline risk–recalibrated	0.9878	0.9876 to 0.9880	0.9570	0.9563 to 09576

As described in the Methods section, ‘Baseline risk–original model’ is the value reported in the original KFRE. ‘Baseline risk–recalibrated’ is the baseline risk value when the model was recalibrated to the current cohort’s data.

ESRD, end stage renal disease; KFRE, Kidney Failure Risk Equation.

### Model calibration and recalibration

**Fig 2:**
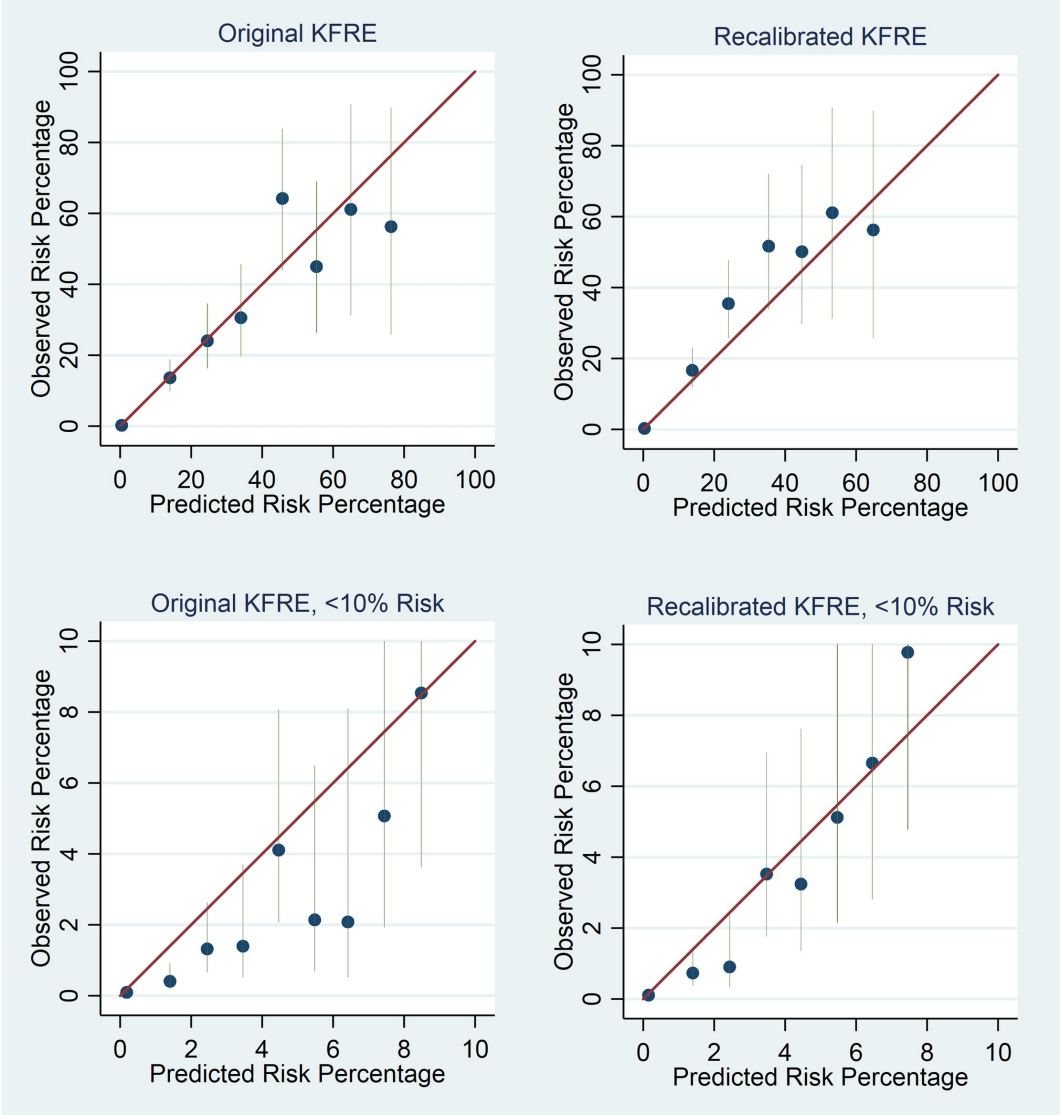
Calibration plots of expected versus observed ESRD events for risk groups for 2-year risk. Top left: original non–North American KFRE calibrated model. Bottom left: detailed plot for risk < 10% for original non–North American KFRE calibrated model. Top right: recalibrated KFRE model. Bottom right: detailed plot for risk < 10% for recalibrated KFRE model. Blue dots represent point estimates, and green vertical lines 95% CIs, for risk groups. Risk groups are split into deciles for the top plots and 1%-wide risk groups for the detailed plots. Fewer than 10 groups are shown in the top plots because no individuals were included in some decile risk groups. ESRD, end stage renal disease; KFRE, Kidney Failure Risk Equation.

**Fig 3:**
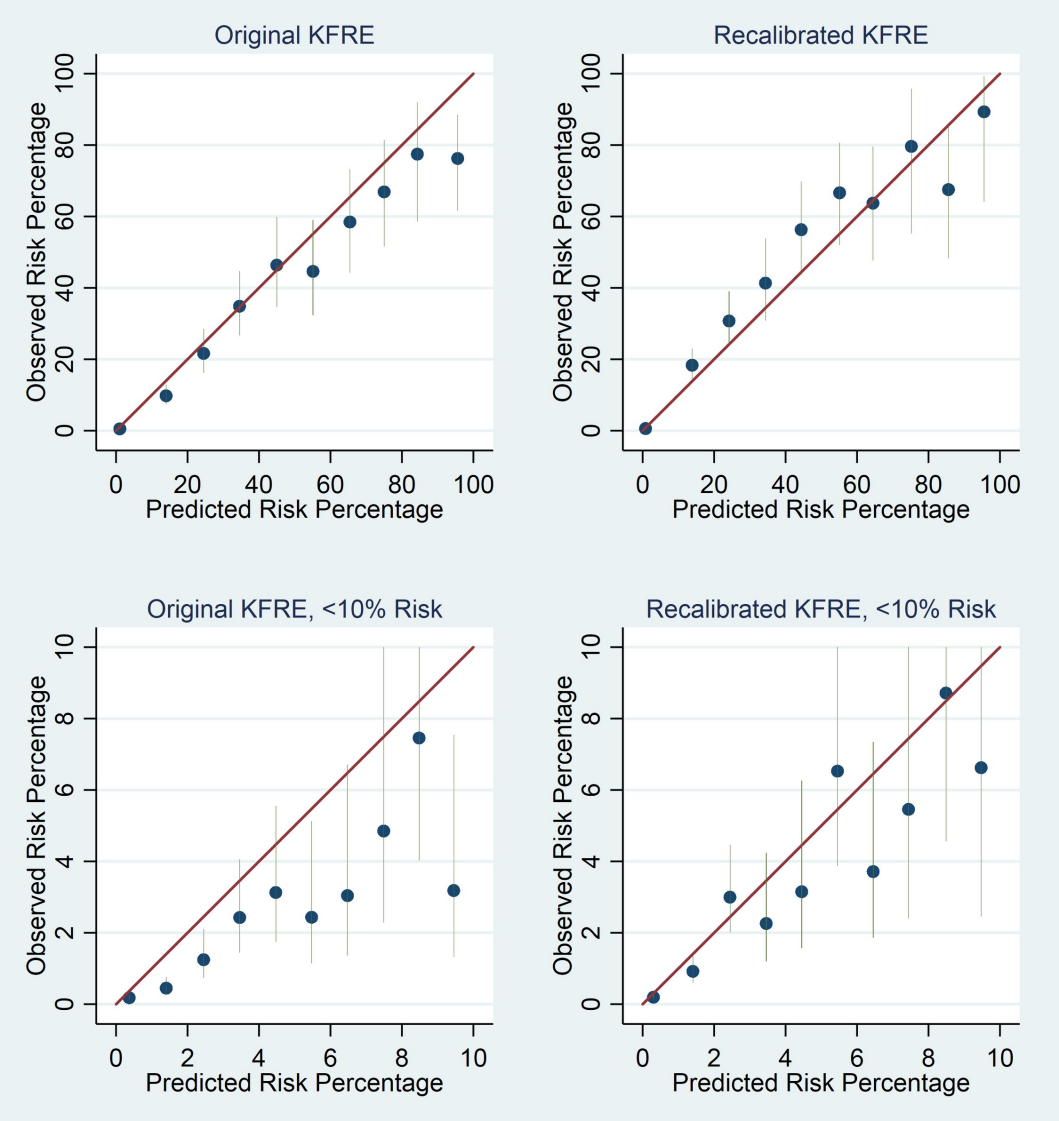
Calibration plots of expected versus observed ESRD events for risk groups for 5-year risk. Top left: original non–North American KFRE calibrated model. Bottom left: detailed plot for risk < 10% for original non–North American KFRE calibrated model. Top right: recalibrated KFRE model. Bottom right: detailed plot for risk < 10% for recalibrated KFRE model. Blue dots represent point estimates, and green vertical lines 95% CIs, for risk groups. Risk groups are split into deciles for the top plots and 1%-wide risk groups for the detailed plots. ESRD, end stage renal disease; KFRE, Kidney Failure Risk Equation.

Calibration plots for the KFRE are shown in Fig 2 for the original 2-year non–North American calibrated model and for an adjusted model based on the current data. Fig 3 shows similar plots for 5-year ESRD prediction. The baseline survival increased from 0.9832 to 0.9878 for the 2-year model and from 0.9365 to 0.9570 for the 5-year prediction model. Calibration plots for the original model suggested overprediction of risk, particularly in lower risk groups for 5-year ESRD prediction. Recalibrated models had improved risk prediction in lower risk groups at the cost of some underprediction of risk in higher risk groups. Underprediction in these groups was less in the 5-year model than in the 2-year model.

### Model sensitivity analysis

We also performed sensitivity analysis using eGFR < 10 ml/min/1.73 m^2^ (eGFR<10), instead of coded ESRD, as the model’s outcome and for different risk group definitions. The eGFR<10 outcome was tested using both a single eGFR < 10 ml/min/1.73 m^2^ and more than 1 eGFR < 10 ml/min/1.73 m^2^ as the definition. The single eGFR<10 definition identified more events, 182 and 483 events respectively at 2 and 5 years, than the coded outcome definition. Discrimination for both the 2-year (C-statistic 0.908, 95% CI 0.883 to 0.934) and 5-year (C-statistic 0.887, 95% CI 0.870 to 0.904) models was lower than for the coded definition. The recalibrated model using the single eGFR<10 definition had a similar recalculated baseline survival to the coded definition, 0.9874 and 0.9506 for the 2- and 5-year models, respectively. Use of the more than 1 eGFR<10 definition reduced the number of events identified compared to the coded event definition and subsequently increased the recalibrated baseline survival for both models. S2 and S3 Tables present the results of this part of the sensitivity analysis. The use of different definitions for risk group assessment of calibration suggested no difference to the findings of the original calibration assessment presented above (S1–S3 Figs).

### Risk re-classification

Table 3 describes the number of individuals eligible for referral (“eligibility assessment cohort”) and overall model performance using current NICE, previously proposed [14,16], and updated criteria. Overall, 1,247 (7.3%) out of 17,077 individuals from the 3 Leicester-based CCGs had been previously seen by secondary care renal services. Using the original KFRE and adopting a ≥3% risk of ESRD over 5 years as the criterion for referral to secondary care increased initial referral eligibility by 84.3% compared to reliance on current NICE referral guidelines. The recalibrated model with referral criterion of ≥3% led to a rise in referral eligibility by 23.3% compared to NICE criteria, with the same number of ESRD cases eligible for referral at baseline. Eligible referrals identified using the KFRE were more likely to have a lower ACR and eGFR.

**Table 3:** Five-year ESRD risk prediction sensitivity, specificities, number of eligible referrals based on criteria (‘baseline referrals’), ESRD cases not meeting eligibility criteria at baseline (‘ESRD not referred at baseline’), and mean characteristics of referrals for different referral criteria.

Measure	NICE Criteria	Recalibrated KFRE			Hybrid criteria	
		≥3%	≥5%	≥15%	KFRE ≥ 5% or eGFR < 30 ml/min/1.73 m^2^	KFRE ≥ 5% or ACR ≥ 70 mg/mmol
Sensitivity	5.5% (4.1 to 7.2)	4.4% (3.3 to 5.8)	6.8% (5.0 to 9.1)	16.7% (11.0 to 23.8)	5.2% (3.8 to 7.0)	6.6% (5.0 to 8.5)
Specificity	99.7% (99.6 to 99.8)	99.7% (99.6 to 99.8)	99.7% (99.6 to 99.8)	99.6% (99.5 to 99.7)	99.7% (99.6 to 99.8)	99.8% (99.7 to 99.8)
Baseline referrals	879 (5.6%)	1,084 (6.9%)	615 (3.8%)	144 (0.9%)	803 (5.1%)	836 (5.3%)
ESRD not referred at baseline	41 (46.1%)	41 (46.1%)	47 (52.8%)	65 (73.0%)	47 (52.8)	34 (38.2%)
Mean age if eligible, years	76.3 (75.4 to 77.2)	76.3 (75.5 to 77.1)	75.2 (74.1 to 76.3)	70.3 (67.6 to 72.9)	77.3 (76.3 to 78.2)	73.8 (72.9 to 74.8)
Female referrals	58.4% (55.1 to 61.6)	55.9% (52.9 to 58.9)	54.3% (50.4 to 58.3)	47.2% (39.0 to 55.4)	59.4% (56.0 to 62.8)	52.8% (49.4 to 56.1)
Mean referral eGFR, ml/min/1.73 m^2^	32.7 (31.9 to 33.5)	30.5 (30.0 to 30.9)	27.7 (27.1 to 28.3)	21.6 (20.5 to 22.7)	27.5 (27.1 to 28.0)	34.0 (33.1 to 34.9)
Mean referral ACR, mg/mmol	77.3 (69.7 to 84.9)	50.6 (45.0 to 56.2)	64.8 (56.2 to 73.4)	130.8 (103.4 to 158.2)	50.5 (43.7 to 57.3)	86.0 (78.3 to 93.8)

Sensitivity refers to the percentage of patients referred who developed ESRD within 5 years of follow-up. Specificity refers to the percentage of patients not initially referred who did not go on to develop ESRD. Figures in parentheses for ‘baseline referrals’ and ‘ESRD not referred at baseline’ refer to percentage of all individuals in cohort not previously seen in renal secondary care. All other figures in parentheses refer to 95% confidence intervals. *n =* 15,830 for eligibility assessment cohort, with 89 ESRD cases.

ACR, albumin-to-creatinine ratio; eGFR, estimated glomerular filtration rate; ESRD, end stage renal disease; KFRE, Kidney Failure Risk Equation; NICE, National Institute of Health And Clinical Excellence.

Based on current NICE referral guidance for CKD, 18 (95% CI 14 to 25) patients would need referral at baseline to identify 1 individual destined to develop ESRD in the subsequent 5 years. Using a recalibrated KFRE ≥ 3% risk of ESRD as the referral criterion resulted in a similar number needed to refer (NNTR) (23 individuals, 95% CI 17 to 31). The NNTR using a risk criterion of ≥5% was 15 (95% CI 11 to 20), with 6 additional patients not eligible for referral at baseline eventually going on to develop ESRD. Referral eligibility was reduced by 30%, and there was no difference in age at referral (*p =* 0.13), but eGFR and ACR were both lower. There was no difference between criteria in the proportion of men and women eligible for referral.

Given these findings, exploratory analysis was performed using alternative referral criteria. A ≥4% criterion reduced referral eligibility by 9.8%, but 3 additional eventual ESRD cases were not referred at baseline compared to current NICE criteria. Hybrid referral criteria of KFRE ≥ 5% and/or the current NICE eGFR criterion (<30 ml/min/1.73 m^2^) did not detect any additional cases of ESRD but did increase referrals by 30.5% compared to the KFRE alone. KFRE ≥ 5% with the current NICE ACR criterion (≥70 mg/mmol) detected an additional 7 cases of ESRD and reduced referral eligibility by 4.9%. Individuals eligible for referral using these criteria were younger (absolute difference 2.4 years, 95% CI 1.1 to 3.8 years, *p <* 0.001), were more likely to be male (absolute difference 5.6%, 95% CI 0.9% to 10.3%, *p =* 0.019), and had a higher eGFR (absolute difference 1.3 ml/min/1.73 m^2^, 95% CI 0.1 to 2.5, *p =* 0.039) than those referred under current NICE criteria. There was no difference in ACR levels at referral (absolute difference 8.7 mg/mmol, 95% CI –2.1 to 19.5, *p =* 0.11). NNTR using KFRE ≥ 5% and/or ACR ≥ 70 mg/mmol was 15 (95% CI 12 to 20). Similar results for referrals were found if analysis was restricted to individuals less than 80 years old.

## Discussion

The KFRE was developed using data from a large multinational individual participant data meta-analysis [14] where a 4-variable equation including sex, age, eGFR, and ACR had excellent discrimination for ESRD events. However, there was clear variation in the baseline risk, and a Non–North American recalibration factor was suggested [14].

Independent external validation of risk scores prior to clinical implementation is rarely performed, and impact studies are even rarer [12,23]. Further, time to event risk models are less likely to be independently externally validated because full model information is rarely presented, including the baseline risk, with the additional issue of some individuals being censored for the outcome [11].

The current independent external validation and clinical impact projection study of the KFRE confirms the findings from the development cohort in relation to discrimination and calibration. Furthermore, these data confirm the temporal and geographical validity of the KFRE in contemporary UK primary care. Discrimination was again found to be excellent for prediction of ESRD within both 2 and 5 years. Nonetheless, it was reasoned that recalibration may be required for routine use of the KFRE in UK primary care. This requirement was exemplified by the need for further adjustment of the baseline risk to improve calibration in the current study. The necessity for KFRE recalibration is explained by several factors. First, CKD in primary care may be less progressive than CKD associated with primary renal diseases, such as glomerulonephritis, likely to be encountered in secondary care. Second, the more contemporary population in the current cohort, compared to the KFRE development cohort established in the 1990s and early 2000s [14], is more likely to have a lower baseline risk. This reduction may be due to population-wide improved management of diabetes and hypertension, and the associated increased use of renin-angiotensin-blocking medications in the UK since the early 2000s [24,25]. Third, the high event rate for all-cause mortality in the current cohort may have affected the estimation of baseline risk for ESRD due to the competing risk from all-cause mortality. This methodological issue has been partially addressed in CKD stages 4 and 5 by the Chronic Kidney Disease Prognosis Consortium through the development of a multi-state model for mortality, ESRD, and cardiovascular events [26].

The clinical impact projection element of the study focused on different recommendations for the KFRE calculated risk level, compared to current NICE guidelines [19], for secondary care CKD referral. NICE is currently considering the use of the KFRE in updated CKD guidelines [15]. Using the recalibrated KFRE alone led to either additional eventual ESRD cases not being eligible for referral at the study’s baseline or large increases in the proportion of the CKD population referred, thus representing no improvement from the current NICE recommendations. A hybrid approach of referral using a recalibrated KFRE criterion of 5-year ESRD risk ≥ 5% and/or ACR ≥ 70 mg/mmol, part of the current NICE criteria, led to additional ESRD cases being detected, whilst also decreasing potential referrals to secondary care. In addition, the individuals identified for referral using these hybrid criteria were more likely to be younger and to have a higher baseline eGFR. As the KFRE was developed in individuals with eGFR < 60 ml/min/1.73 m^2^, the hybrid criteria would also continue to suggest referral of individuals with significant proteinuria regardless of their eGFR. However, the hybrid criteria have only undergone internal validation in the current cohort. They will therefore require further testing of validity, including in non-UK populations, as currently there may statistical overfitting of the data, leading to overly optimistic estimates of the criteria’s benefits.

The cost of initial attendance to secondary care renal services, updated to 2019 costs, for CKD has been estimated at £318, with annual ongoing costs of approximately £534 [19]. NHS England 2010 costs for non-ESRD CKD outpatient appointments have been estimated at £53 million per year [5]. Therefore, use of the hybrid referral criteria (KFRE ≥ 5% or ACR ≥ 70 mg/mmol), as described in Table 3, to reduce referral eligibility by approximately 5% could potentially lead to large cost savings across the UK healthcare system. Safely delaying referrals may also decrease costs, provided that there is not a consequential effect of later, and more costly, referrals in those who do go on to develop ESRD. The implementation of these criteria can be performed across a whole general practice through the use of electronic health records. Risk prediction models, such as QRisk2 and QRisk3 for primary prevention of cardiovascular disease, have already been implemented by electronic healthcare record companies into standard general practice software in the UK and other countries. Therefore, potential implementation financial and time costs to individual general practices are likely to be minimal. Overall, a recalibrated-KFRE-driven risk-based approach with the addition of the ACR-based criterion [19] for referral may lead to more appropriate referrals to secondary care, conserving resources and reducing for patients the associated psychological stress of referral. Further, the use of the ACR criterion will continue to highlight for referral individuals with preserved eGFR but significant proteinuria, often younger individuals with primary renal disease whose lifetime ESRD risk remains substantially elevated.

The original KFRE development included approximately 15,000 European CKD patients. Therefore, the current study extends the validity of KFRE use to UK and European primary care populations. In addition, the study of referrals is critical for resource allocation in universal healthcare systems, such as those in the majority of European countries.

There are a number of strengths to the current study. First, primary care data were used, and therefore the results provide, to our knowledge for the first time, evidence that the KFRE can be reliably implemented in routine CKD clinical care by general practitioners. Second, the cohort presented is also more contemporary than those used to develop the KFRE. Only 4 of the 31 cohorts contributing to the original KFRE development included baseline data from 2010 onwards [14]. None of these were from the UK. Third, ESRD events were identified from linkage of both primary and secondary care records. Although identification and linking of data for ESRD events in primary and secondary care has not been studied, data from cardiovascular disease would suggest that data from both primary and secondary care are required to identify the optimal number of events [27]. Linkage of data also identified individuals already known to secondary care services at baseline. These individuals could then be excluded when potential referral patterns based on predicted risk were studied. This issue of risk score impact is very rarely studied [12]. Fourth, whilst ESRD is a relatively rare event, even in a CKD population, the size of the current cohort allowed identification of more than 200 ESRD events, a figure suggested to be required to allow consistency of results in external validation [20].

The study also has some weaknesses. Whilst the cohort is representative of an adult population of over 600,000 individuals, geographically it was restricted to the East Midlands and therefore may not be a nationally representative sample. In addition, the secondary care ESRD event data were the regional data provided to the UK Renal Registry and therefore were not national outcome data, so they may not have identified all ESRD events for individuals in the cohort. However, these events may have been identified through the recorded ESRD cases from the primary care data. We were unable to quantify the effect this may have had on identification of ESRD events. The current study also assumes that referrals to secondary care renal services only occur to manage the risk of ESRD and not for management of other conditions, such as CKD-related anaemia and mineral bone disease. In addition, referral to secondary care should not be solely based on KFRE predicted risk if primary renal conditions, such as inherited conditions (e.g., polycystic kidney disease) or glomerulonephritis, are suspected. This study does not provide new evidence for referral in these conditions. The current guidelines including criteria based on haematoproteinuria and a family history of ESRD should not be changed, as the KFRE alone is unlikely to be an accurate predictor if these conditions are suspected. The KFRE also only predicts outcomes for up to 5 years and so does not consider longer, including lifetime, risk of ESRD. Longer time spans are particularly relevant for younger individuals with typically heavier proteinuria, and these individuals will continue to be detected by the suggested hybrid criteria.

The cohort used for the study was also not a specific research study dataset, but was based on routinely collected data. This can be perceived as both a strength and a weakness of the study. As the aim was to assess the KFRE in routine clinical CKD care, using a clinical practice dataset is probably the most suitable approach for this. However, clinical datasets do have weaknesses. For the KFRE, this is probably most true in relation to the completeness of data for ACR. It is likely that some subgroups, such as those with diabetes mellitus, are overrepresented in the cohort, and some underrepresented, such as frailer, older individuals.

In this study, we have independently externally validated the 2- and 5-year KFRE and shown that it has excellent discrimination, but requires model recalibration, for ESRD event prediction in UK primary care. Our findings suggest that application of a recalibrated KFRE, in combination with an ACR-based criterion, in UK primary care may reduce unnecessary referrals to secondary care, thus reducing healthcare costs and patient anxiety, without increasing the risk of missing patients who will go on to develop ESRD. However, further external validation in more diverse cohorts of the suggested referral criteria, including in non-UK cohorts, is required before they can be implemented in clinical care.

## Supporting information

S1 Data Data as CSV file to reproduce the analysis. (CSV)

S1 Fig Calibration plots of 5-year expected versus observed events by risk groups with equal numbers of events per group. (DOCX)

S2 Fig Calibration plots of 5-year expected versus observed events by 2%-wide predicted risk groups calculated by the original KFRE, up to 20% risk. (DOCX)

S3 Fig Calibration plots of 5-year expected versus observed events by <3%, 3% to <5%, 5% to <15%, 15% to <25%, 25% to <50%, and ≥50% risk groups [14]. (DOCX)

S1 Table TRIPOD checklist. (DOCX)

S2 Table Sensitivity analysis for event definition based on eGFR. (DOCX)

S3 Table Overlap of events by ESRD definition. (DOCX)

S1 Text The 4-variable KFRE. (DOCX)

S2 Text Variable names for S1 Data. (DOCX)

## Abbreviations

ACR: albumin-to-creatinine ratio
CCG: Clinical Commissioning Group
CKD: chronic kidney disease
CKD-EPI: Chronic Kidney Disease Epidemiology Collaboration
eGFR: estimated glomerular filtration rate
ESRD: end stage renal disease
KFRE: Kidney Failure Risk Equation
NHS: National Health Service
NICE: National Institute of Health And Clinical Excellence
NNTR: number needed to refer
