# Correction: The Kidney Failure Risk Equation for prediction of end stage renal disease in UK primary care: An external validation and clinical impact projection cohort study

**DOI:** 10.1371/journal.pmed.1003313

**Published:** 2020-07-24

**Authors:** Rupert W. Major, David Shepherd, James F. Medcalf, Gang Xu, Laura J. Gray, Nigel J. Brunskill

Following the review of the open source data file mentioned in the Data Availability Statement (S1 Data), there are discrepancies with some of the variables provided in the open source data, meaning that the results in the published manuscript cannot be re-produced from the open source data file. The data available on Figshare has been updated.

The Data Availability Statement should therefore read: Data to reproduce the analysis are available on Figshare (https://doi.org/10.25392/leicester.data.9860807.v1).
