# Supplementary material for: The Kidney Failure Risk Equation for prediction of end stage renal disease in UK primary care: An external validation and clinical impact projection cohort study
Source: PLoS Med. 2019 Nov 6;16(11):e1002955. doi: 10.1371/journal.pmed.1002955 (PMC6834237; doi:10.1371/journal.pmed.1002955)
Supplement: S1 Fig — (DOCX) [file pmed.1002955.s002.docx]

**Supporting Information – ‘The Kidney Failure Risk Equation for prediction of end stage renal disease in UK primary care: an external validation and clinical impact projection cohort study’**

**Supporting Information Figure 1 –** Calibration plots of 5 year expected versus observed events by groups with equal number of events per group. A (top left) – original ‘Non-North American’ calibrated model, B (top right) – detailed plot for risk <20% for original ‘Non-North American’ calibrated model, C (bottom left) – re-calibrated model, D (bottom right) – detailed plot for risk <20% for re-calibrated model. Blue dots represent point estimates and green vertical lines 95% CI for risk groups.

**
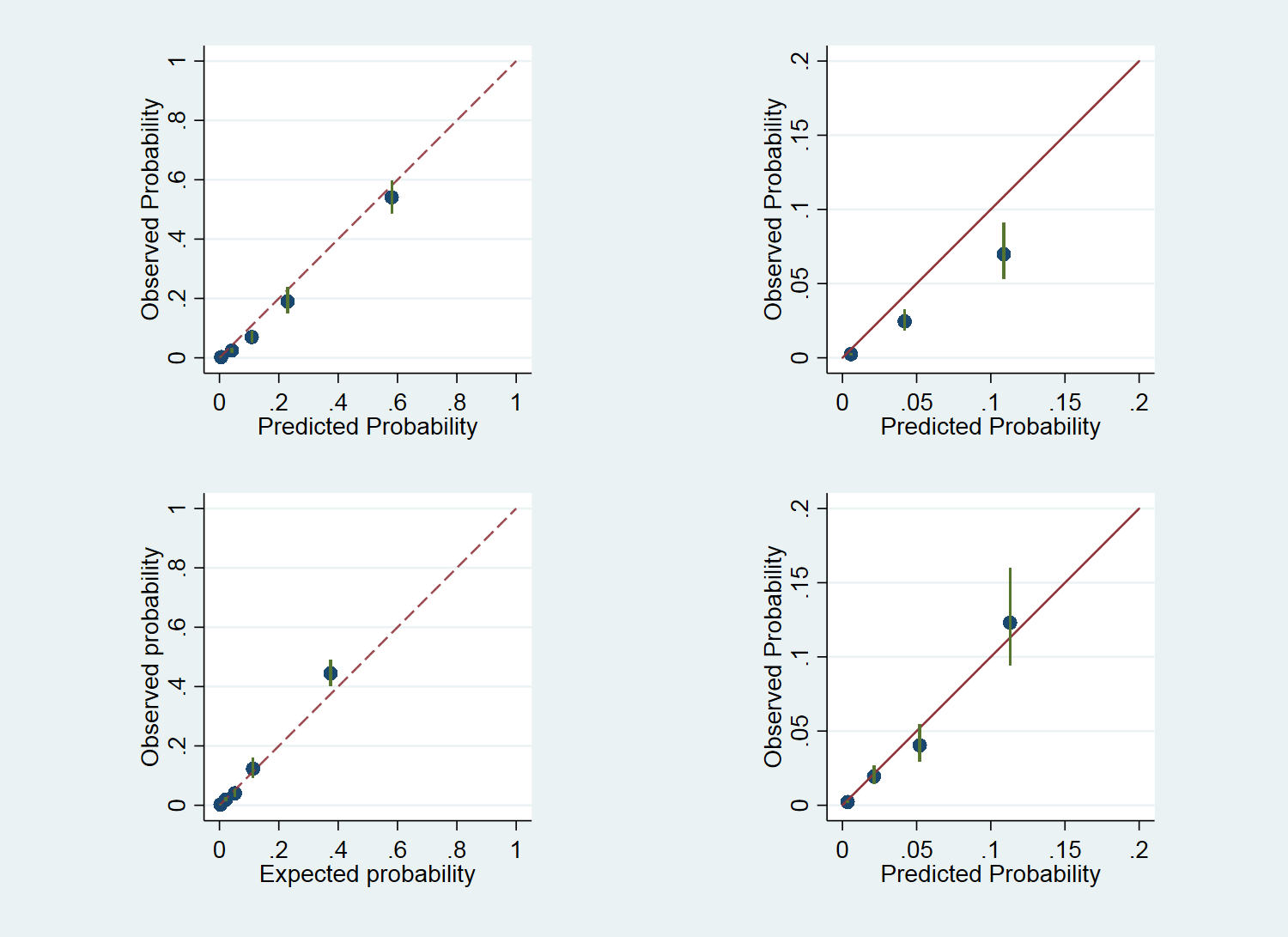

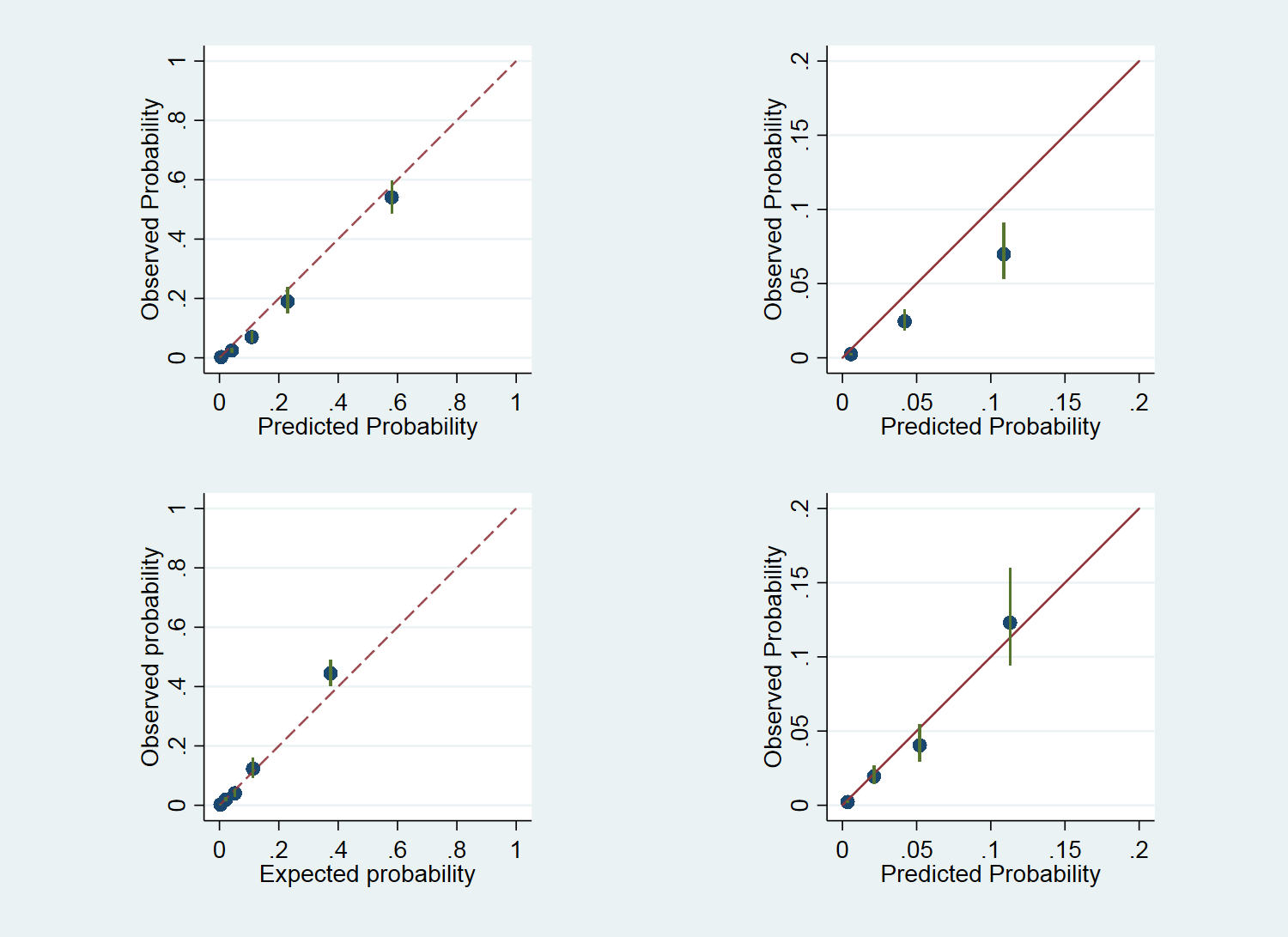
**
