# Supplementary material for: The Kidney Failure Risk Equation for prediction of end stage renal disease in UK primary care: An external validation and clinical impact projection cohort study
Source: PLoS Med. 2019 Nov 6;16(11):e1002955. doi: 10.1371/journal.pmed.1002955 (PMC6834237; doi:10.1371/journal.pmed.1002955)
Supplement: S2 Fig — (DOCX) [file pmed.1002955.s003.docx]

**Supporting Information – ‘The Kidney Failure Risk Equation for prediction of end stage renal disease in UK primary care: an external validation and clinical impact projection cohort study’**

**Supporting Information Figure 2** – Calibration plots of 5 year expected versus observed events by groups split into 2% wide predicted risk groups calculated by the original KFRE, upto 20% risk. A (left) – detailed plot for risk <25% for original ‘Non-North American’ calibrated model, B (right) – detailed plot for risk <25% for re-calibrated model. Blue dots represent point estimates and green vertical lines 95% CI for risk groups.

**
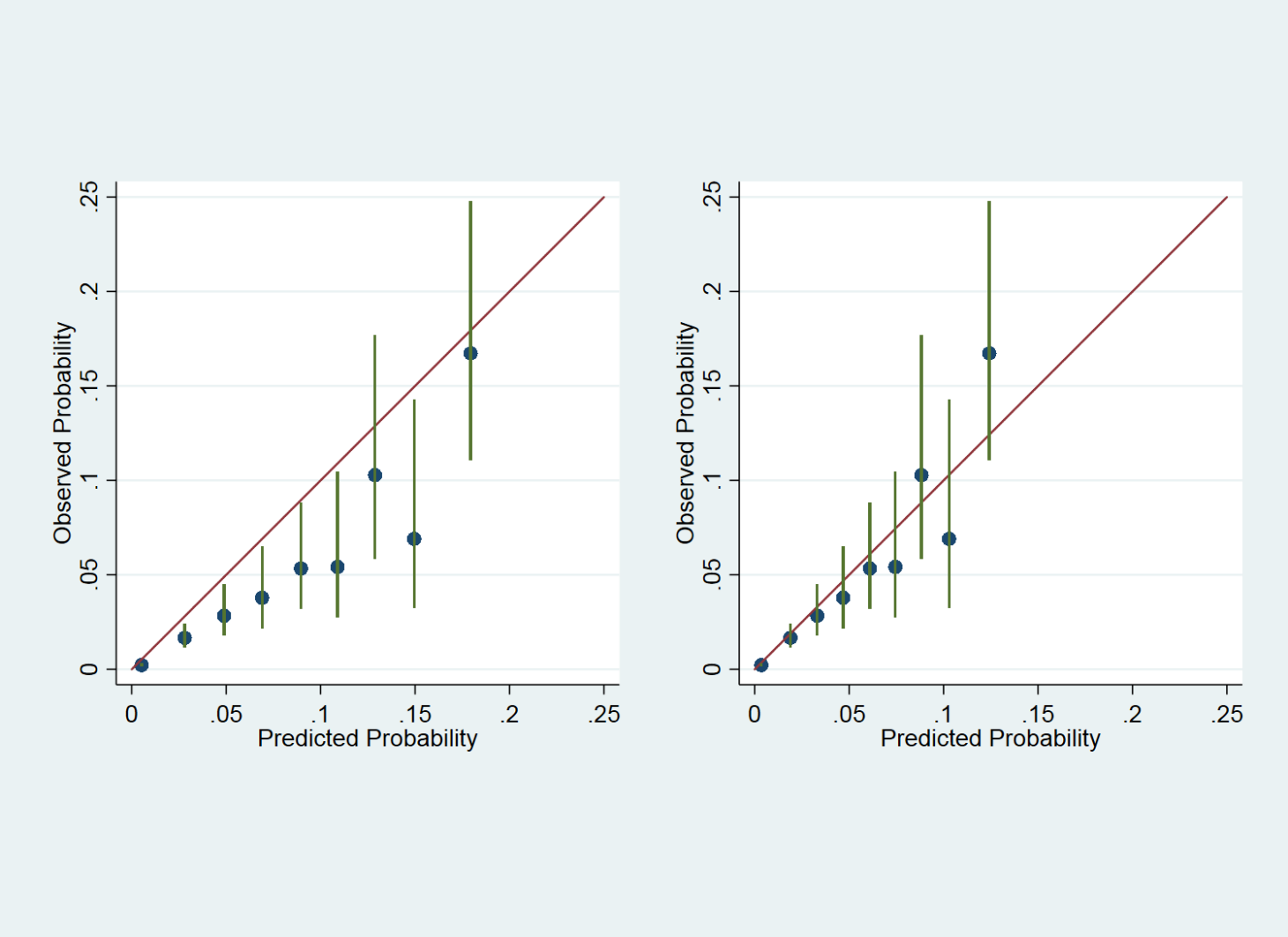
**
