# Supplementary material for: The Kidney Failure Risk Equation for prediction of end stage renal disease in UK primary care: An external validation and clinical impact projection cohort study
Source: PLoS Med. 2019 Nov 6;16(11):e1002955. doi: 10.1371/journal.pmed.1002955 (PMC6834237; doi:10.1371/journal.pmed.1002955)

**Supporting Information – ‘The Kidney Failure Risk Equation for prediction of end stage renal disease in UK primary care: an external validation and clinical impact projection cohort study’**

**Supporting Information Figure 3** – Calibration plots of expected versus observed ESRD events for risk groups for 5 year risk. A (top left) – original ‘Non-North American’ KFRE calibrated model, B (top right) – detailed plot for risk <10% for original ‘Non-North American’ KFRE calibrated model, C (bottom left) – re-calibrated KFRE model, D (bottom right) – detailed plot for risk <10% for re-calibrated KFRE model. Blue dots represent point estimates and green vertical lines 95% CI for risk groups. Risk groups are split into <3%, 3-5%, 5-15%, 15-25%, 25-50% and ≥50% risk.


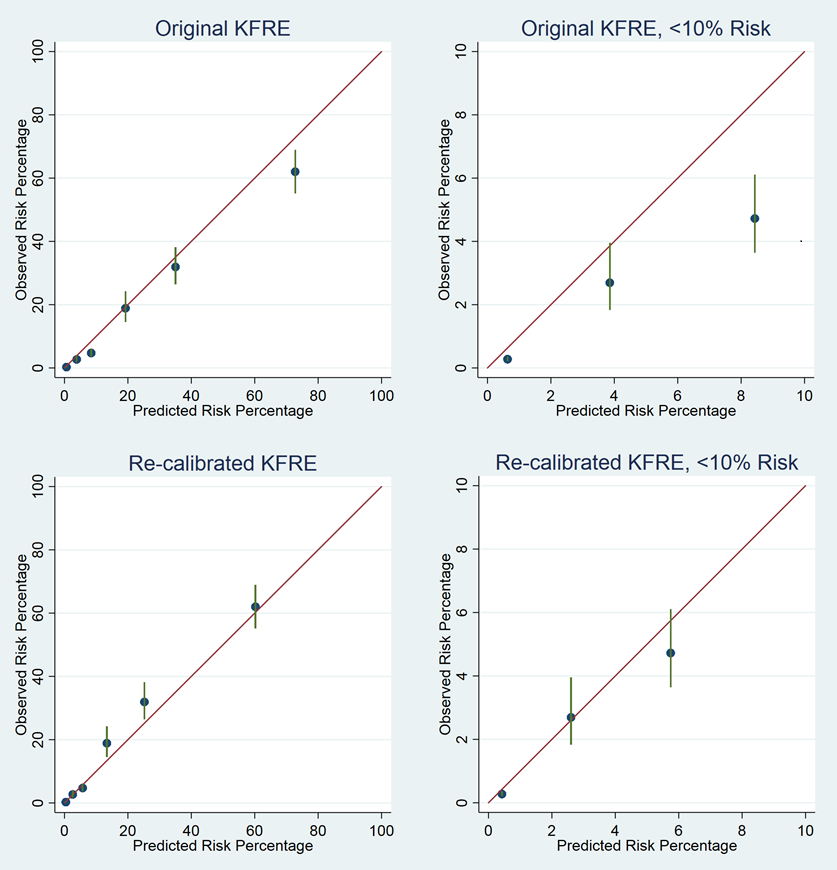

Supplement: S3 Fig — (DOCX) [file pmed.1002955.s004.docx]
