# Supplementary material for: The Kidney Failure Risk Equation for prediction of end stage renal disease in UK primary care: An external validation and clinical impact projection cohort study
Source: PLoS Med. 2019 Nov 6;16(11):e1002955. doi: 10.1371/journal.pmed.1002955 (PMC6834237; doi:10.1371/journal.pmed.1002955)
Supplement: S2 Table — (DOCX) [file pmed.1002955.s006.docx]

**Supporting Information – ‘The Kidney Failure Risk Equation for prediction of end stage renal disease in UK primary care: an external validation and clinical impact projection cohort study’**

**Supporting Information Table 2** – Sensitivity analysis for event definition based on eGFR.

| **Model** | **Metric** | **Tangri et al** | **Coded Definition** | **1 eGFR<10** | | **>1 eGFR <10** | |
| --- | --- | --- | --- | --- | --- | --- | --- |
| 2 Years | Events |  | 176 | 182 |  | 115 |  |
|  | C Statistic |  | 0.933 | 0.9082 | 0.8826 to 0.9339 | 0.9528 | 0.9310 to 0.9746 |
|  | Baseline Risk | 0.9832 | 0.9878 | 0.9874 | 0.9872 to 0.9876 | 0.9920 | 0.9919 to 0.9921 |
| 5 Years | Events |  | 429 | 483 |  | 286 |  |
|  | C Statistic |  | 0.926 | 0.8867 | 0.8697 to 0.9036 | 0.9389 | 0.9236 to 0.9541 |
|  | Baseline Risk | 0.9365 | 0.9570 | 0.9506 | 0.9498 to 0.9513 | 0.9710 | 0.9705 to 0.9714 |
