# Supplementary material for: The Kidney Failure Risk Equation for prediction of end stage renal disease in UK primary care: An external validation and clinical impact projection cohort study
Source: PLoS Med. 2019 Nov 6;16(11):e1002955. doi: 10.1371/journal.pmed.1002955 (PMC6834237; doi:10.1371/journal.pmed.1002955)
Supplement: S3 Table — (DOCX) [file pmed.1002955.s007.docx]

**Supporting Information – ‘The Kidney Failure Risk Equation for prediction of end stage renal disease in UK primary care: an external validation and clinical impact projection cohort study’**

**Supporting Information Table 3** – Overlap of events by ESRD definition.

| **Definition** | **No eGFR<10** | **1 eGFR<10** |  | **≤1 eGFR <10** | **>1 eGFR <10** |
| --- | --- | --- | --- | --- | --- |
| Not Coded ESRD | 34,900  (98.2%) | 210  (0.59%) |  | 35,038  (98.59%) | 72  (0.20%) |
| Coded ESRD | 156  (0.44%) | 273  (0.77%) |  | 215  (0.60%) | 214  (0.60%) |
