# Supplementary material for: The Kidney Failure Risk Equation for prediction of end stage renal disease in UK primary care: An external validation and clinical impact projection cohort study
Source: PLoS Med. 2019 Nov 6;16(11):e1002955. doi: 10.1371/journal.pmed.1002955 (PMC6834237; doi:10.1371/journal.pmed.1002955)
Supplement: S1 Text — (DOCX) [file pmed.1002955.s008.docx]

**Supporting Information Text 1– ‘The Kidney Failure Risk Equation for prediction of end stage renal disease in UK primary care: an external validation and clinical impact projection cohort study’**

**Kidney Failure Risk Equation 4-Variable Equation**

The risk of ESRD using the KFRE 4-variable equation calibrated to a non-North American population is calculated as:

For five year risk: 1 - 0.9365^exp(βsum)^

For two year risk: 1 - 0.9832^exp(βsum)^

Where βsum = -0.2201 × (age/10 – 7.036)

+ 0.2467 × (male – 0.5642)

– 0.5567 × (eGFR/5 – 7.222)

+ 0.4510 × (logACR – 5.137)
