# Supplementary material for: The Kidney Failure Risk Equation for prediction of end stage renal disease in UK primary care: An external validation and clinical impact projection cohort study
Source: PLoS Med. 2019 Nov 6;16(11):e1002955. doi: 10.1371/journal.pmed.1002955 (PMC6834237; doi:10.1371/journal.pmed.1002955)
Supplement: S2 Text — (DOCX) [file pmed.1002955.s009.docx]

**Supporting Information – ‘The Kidney Failure Risk Equation for prediction of end stage renal disease in UK primary care: an external validation and clinical impact projection cohort study’**

**Supporting Information Text 2** – Variable Names for S1 Data

Data to reproduce the results for the manuscript are provided in ‘kfre_plosmed_anon.csv’.

The variables are as follows:

id – anonymised numeric id

age - age in years at baseline

epi_egfr - EPI eGFR at baseline

death - binary outcome of death during follow-up

female – binary gender variable, “1”=female “0”=male

esrd – endstage renal failure event during follow-up

acr_mgmmol = ACR at baseline measured in mg/mmol

time = duration in study in days

co-morbidities – “1”=present at baseline, “0”=not present at baseline

dm = diabetes mellitus – either type 1 or type 2

hf = heart failure

cvd = cardiovascular disease

htn = hypertension

neph_known – status of secondary care renal referral at baseline – “1”=known to secondary care, “0”=not known to secondary care, “-“=unknown status
